# Things must not fall apart: the ripple effects of the COVID-19 pandemic on children in sub-Saharan Africa

**DOI:** 10.1038/s41390-020-01174-y

**Published:** 2020-09-24

**Authors:** Modupe Coker, Morenike O. Folayan, Ian C. Michelow, Regina E. Oladokun, Nguavese Torbunde, Nadia A. Sam-Agudu

**Affiliations:** 1 International Research Center of Excellence, Institute of Human Virology Nigeria, Plot 252 Herbert Macaulay Way, Abuja, Nigeria; 2 Department of Epidemiology, Geisel School of Medicine at Dartmouth, Lebanon, NH USA; 3 Department of Oral Biology, School of Dental Medicine, Rutgers University, Newark, NJ USA; 4 Department of Child Dental Health, Obafemi Awolowo University, Ile-Ife, Nigeria; 5 Department of Pediatrics, Division of Infectious Diseases, Alpert Medical School of Brown University and Center for International Health Research, Rhode Island Hospital, Providence, RI USA; 6 Department of Paediatrics, College of Medicine University of Ibadan and University College Hospital, Ibadan, Nigeria; 7 Pediatric and Adolescent HIV Unit, Institute of Human Virology Nigeria, Abuja, Nigeria; 8 Institute of Human Virology and Department of Pediatrics, University of Maryland School of Medicine, Baltimore, MD USA; 9 Department of Paediatrics, University of Cape Coast School of Medical Sciences, Cape Coast, Ghana

## Abstract

**Abstract:**

Zero to 19 year-old children in sub-Saharan Africa bear a disproportionate proportion of the global burden of communicable and non-communicable diseases. Significant public health gains have been made in the fight against these diseases, however, factors such as underequipped health systems, disease outbreaks, conflict, and political instability continue to challenge prevention and control. The novel coronavirus disease (COVID-19) pandemic caused by severe acute respiratory syndrome coronavirus 2 (SARS-CoV-2) introduces new challenges to public health programs in sub-Saharan Africa. Of particular concern are programs targeting major conditions among children, such as undernutrition, vaccine-preventable pneumonia and diarrhea, malaria, tuberculosis, HIV, and sickle cell disease. This article focuses on the impact of the COVID-19 pandemic on child health in sub-Saharan Africa. We review the epidemiology of major pediatric diseases and, referencing modeling projections, discuss the short- and long-term impact of the pandemic on major disease control. We deliberate on potential complications of SARS-CoV-2 co-infections/co-morbidities and identify critical social and ethical issues. Furthermore, we highlight the paucity of COVID-19 data and clinical trials in this region and the lack of child participants in ongoing studies. Lastly, approaches and interventions to mitigate the pandemic’s impact on child health outcomes are discussed.

**Impact:**

- Children in sub-Saharan Africa bear a disproportionate burden of communicable and non-communicable diseases globally; this remains true even as the COVID-19 pandemic persists.
- Amidst the fast-expanding COVID-19 literature, there is little comprehensive coverage of the pandemic’s indirect impact on child health in sub-Saharan Africa.
- This article comprehensively outlines the threat that the pandemic poses to major disease prevention and control for children in sub-Saharan Africa. It discusses the potential impact of SARS-CoV-2 co-infections/co-morbidities, highlights research gaps, and advocates for data and action to mitigate the ripple effects of the pandemic on this population.

## Introduction

As of August 8, 2020, there have been >19 million cases of severe acute respiratory syndrome coronavirus 2 (SARS-CoV-2) infections and over 716,000 deaths (3.7% case fatality rate) reported worldwide,^1^ with 872,501 cases and 16,041 deaths (1.8% case fatality rate) in Africa.^1^ The vast majority of novel coronavirus disease (COVID-19) cases are in adults, with severe manifestations and higher mortality occurring among people over 60 years of age and those with underlying systemic conditions, particularly cardiovascular diseases, diabetes, and chronic pulmonary disorders.^2^ The later arrival of SARS-CoV-2^3,4^ (affording time for better response preparation), and higher proportion of youth under 20 years in sub-Saharan Africa (SSA) (52.7%) compared to Asia (31.2%), North America (24.5%), and Europe (21.2%)^5^ may partly explain the relatively low COVID-19 case burden and case fatality rate in this region. However, testing capacity and coverage is also much lower in SSA than in other regions,^6^ therefore, undercounting is likely contributing to underestimations.

Available data on COVID-19 in children are mostly from China, the United States of America (US), and Europe. These reports indicate that patients 0–19 years account for 1–5% of confirmed cases.^7–10^ The majority of children have a milder disease course, better treatment outcomes, and significantly lower mortality compared to adults.^7–11^ COVID-19 has also been described among pregnant women and neonates, with what appears to be more severe disease among pregnant versus non-pregnant women.^12–15^

The direct and indirect impacts of COVID-19 among children in SSA are yet to be reported or described in detail. In May 2020, the Africa Centres for Disease Control and Prevention reported that children under 15 years constituted only 2.1% of COVID-19 cases in Africa.^16^ Beyond that, there has been little available information on case counts and spectrum of clinical presentation of COVID-19 among African children. Factors including underequipped health/research infrastructure, political denial and misinformation, and ongoing conflict and humanitarian emergencies^17–19^ may underlie suboptimal disease surveillance and mask the true impact of COVID-19 on children in SSA. This is of particular concern, since children in this region bear a significant burden of global infectious disease morbidity and mortality. Social and economic lockdowns have further intensified their vulnerabilities,^20^ including loss of household income, poor access to healthcare services, and other multidimensional impact.^21^ For some African countries, movement restrictions, workplace/school closures, and travel bans started as early as March 2020.^22^

This review highlights the vulnerabilities of children in the context of the COVID-19 pandemic in SSA. For this purpose, the term “children” refers to people 0–19 years old unless otherwise specified. We deliberate on the impact of this novel infectious disease on the prevention and control of other communicable and non-communicable diseases (NCDs) in this population and advocate for continued commitment to fighting these diseases while responding to the pandemic.

## Narrative

### Impact of the COVID-19 pandemic on prevention and control of diseases of public health importance among children in SSA

With respect to morbidity and mortality, the leading infectious diseases among children in SSA are malaria, human immunodeficiency virus (HIV), tuberculosis (TB), and vaccine-preventable diseases, including infectious diarrhea, pneumonia, and meningitis^23^ (Table 1). NCDs are also predominant, including undernutrition (often a comorbidity with infectious diseases) and sickle cell disease (SCD).^24^ The COVID-19 pandemic is expected to significantly impact health programming and initiatives for these major diseases.

**Table 1:** Major communicable and non-communicable diseases affecting children in sub-Saharan Africa (in order of prevalence)^a,b^.

	Disease	Epidemiological data relevant to sub-Saharan Africa		Major strategies and initiatives which could be impacted by the COVID-19 pandemic
		Estimated prevalence per 100,000 children	Estimated deaths per 100,000 children	
*Non-communicable diseases*				
1	Undernutrition	CU5: 32% stunted and 6.2% wasted^25^ CU5 with PEM: 8044 cases 5–14 years with PEM: 613 cases	CU5: 65 (from PEM) 5–14 years: 3 (from PEM)	WHO Global Action Plan on Child Wasting^99^ Africa Regional Nutrition Strategy^100^
2	Sickle cell disease	CU5: 435 cases 5–14 years: 626 cases	CU5: 9 5–14 years: 3	Penicillin prophylaxis, timely routine vaccinations (especially influenza, meningococcal, pneumococcal), hydroxyurea treatment^58^
*Communicable/infectious diseases*				
1	Malaria	CU5: 13,961 cases 5–14 years: 29,677 cases	CU5: 201 5–14 years: 28	WHO Global Technical Strategy for Malaria^36^ WHO Malaria Vaccine Implementation Program^102^
2	Diarrhea	CU5: 3493 cases 5–14 years: 2927 cases	CU5: 205 5–14 years: 14	The Integrated Global Action Plan for Pneumonia and Diarrhea (includes rotavirus vaccine)^95^
3	Meningitis (all causes)	CU5: 315 cases 5–14 years: 871 cases	CU5: 64 5–14 years: 9	WHO Defeating Meningitis by 2030 Road Map,^94^ which includes *H. influenzae* type b, *Neisseria meningitidis*, and pneumococcal vaccines
4	HIV/AIDS	CU5: 259 cases 5–14 years: 1011 cases	CU5: 40 5–14 years: 30	Start Free Stay Free AIDS Free^93^
5	Pneumonia (lower respiratory infections)	CU5: 245 cases 5–14 years: 207 cases	CU5: 253 5–14 years: 12	The Integrated Global Action Plan for Pneumonia and Diarrhea^95^ (includes measles, pertussis, *S. pneumoniae*, and *H. influenzae* type b vaccines)
6	Tuberculosis (all active cases)	CU5: 100 cases 5–14 years: 121 cases	CU5: 26 5–14 years: 5	WHO Roadmap Towards Ending TB in Children and Adolescents^92^
7	Measles	CU5: 71 cases 5–14 years: 11 cases	CU5: 34 5–14 years: 4	Global Measles and Rubella Strategic Plan 2012–2020, Measles and Rubella Initiative, and Measles Outbreak Response (all include measles, mumps, and rubella vaccine)^96^

*CU5* children <5 years of age, *PEM* protein energy malnutrition, *WHO* World Health Organization, *TB* tuberculosis.

^a^Unless otherwise indicated, epidemiological data source is: Global Burden of Disease Collaborative Network. Global Burden of Disease Study 2017 (GBD 2017) results. Institute for Health Metrics and Evaluation, 2018 (http://ghdx.healthdata.org/gbd-results-tool).^23^ Disaggregated data for children only available for those <15 years of age.

^b^Numbered citations in this table correspond to sources in the manuscript’s list of references.

**Fig. 1:**
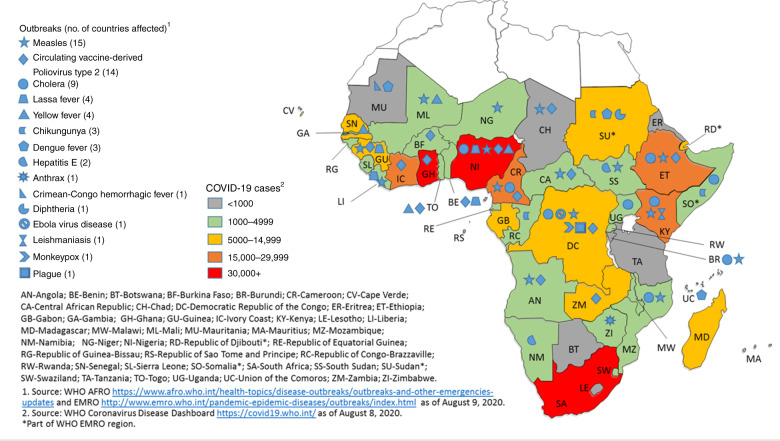
Outbreaks concurrent with the COVID-19 pandemic in sub-Saharan Africa. The blue symbols represent the different infectious diseases causing outbreaks in the region, with number of countries affected by that particular disease outbreak indicated in brackets. The colored key highlights the case burden for COVID-19 in each country.

Figure 1 shows outbreaks of other infectious diseases in the setting of the ongoing COVID-19 pandemic in SSA.

#### Undernutrition

SSA accounts for an estimated 23% of wasted (low weight for age) and 36% of stunted (low height for age) children under 5 years of age (CU5) worldwide.^25^ Wasted children are immunologically compromised and at increased risk of death; stunted children experience learning difficulties and may never achieve full cognitive potential.^26,27^ CU5 mortality rate from protein energy malnutrition in SSA is estimated at 65 per 100,000^23^ (Table 1). However, undernutrition is an underlying factor in many more child deaths from both communicable and NCDs.^25,28^

Children are usually worst affected when there is a reduction in household income and food insecurity, which are anticipated consequences of the COVID-19 pandemic.^29^ Indeed, Headey and colleagues estimate a nearly 15% increase (~6.7 million cases) in the prevalence of moderate or severe wasting and nearly 130,000 additional deaths among CU5 in low- and middle-income countries in 2020 due to COVID-19-related economic losses.^30^ SSA contributes 22% and 52% to the wasting and death estimates, respectively.^30^

Lockdowns with concurrent school closures have also affected access to school-based meals, which for many children, are one of the few consistent sources of food. Thus the pandemic has further exposed children to hunger, poor nutrition, and consequentially negative impacts on cognitive development; all this at a time when many families are dealing with unemployment and income loss.^31^ The World Food Programme estimates that globally 368 million children (47% girls) from pre-primary to secondary school level are currently missing school meals; an estimated 148 million are in SSA.^32–34^ The impact may be worse for girls in SSA, where school meals are often a strong incentive for parents to enroll female children and thereby prevent early child marriage.

#### Malaria

Africa accounts for 93% of malaria cases and 94% of malaria-related deaths worldwide.^35^ Six SSA countries account for more than half of global annual malaria cases caused by *Plasmodium falciparum*, which is responsible for the most prevalent and serious malaria infections.^35^ Thirty-one SSA countries are on track to meet the milestones of the Global Technical Strategy for Malaria 2016–2030, which include reducing malaria case incidence by ≥40% between 2015 and 2020.^36^ However, this hard-earned success is fragile: in addition to emerging drug and insecticide resistance, the COVID-19 pandemic further threatens malaria elimination.^37^ Pandemic responses may result in the scaling back of long-lasting insecticidal net distribution, indoor residual spraying, seasonal malaria chemoprophylaxis campaigns, access to rapid diagnostic tests, and effective malaria treatment. Hogan et al. estimate that pandemic-related disruption of net distribution and other health services will lead to a 36% increase in malaria-related deaths over 5 years in high-burden low- and middle-income countries.^38^ An analysis by the World Health Organization (WHO) predicts an up to 23% increase in malaria-related cases in SSA and up to 102% more deaths, of which 70% would be among CU5.^37^

#### Vaccine-preventable diseases (including diarrhea and pneumonia)

The WHO Expanded Program on Immunization has made significant gains in controlling the 12–15 infectious diseases targeted by routinely recommended immunizations for children.^39^ One of these immunizations is associated with gastrointestinal illness (rotavirus) and five with respiratory diseases (diphtheria, *Haemophilus influenzae* type b, measles, pertussis, and *Streptococcus pneumoniae*).^39^ Diarrhea and acute respiratory infections remain the leading causes of mortality among CU5 worldwide and in SSA: in 2017, children in SSA accounted for 23% of diarrhea-related deaths and 51% of acute respiratory infections.^40^ SSA bears >80% of global rotavirus mortality, at a rate of 67/100,000 population of CU5 versus the global rate of 20/100,000.^41^ Pneumonia-specific mortality in CU5 has declined since the introduction of the *H. influenzae* type b and pneumococcal conjugate vaccines,^42^ and so has the burden of rotavirus-related diarrhea and other vaccine-preventable diseases,^43^ especially in SSA.

As the number of COVID-19 cases rise in SSA, there is concern that immunization access and coverage may be compromised through diversion of limited human, financial, and other resources to the pandemic response.^44,45^ Also, complete or partial lockdowns in several African countries will hinder children’s access to clinics and community-based immunization services,^45,46^ and caregivers may avoid facility immunization visits due to fear of COVID-19 exposure. Abbas et al.’s benefit–risk analysis study reported that, for every one excess death attributable to SARS-CoV-2 infection from exposure, 85,000 deaths could be averted among CU5 in SSA who successfully receive routine vaccinations.^41^ The benefit–risk ratio for CU5 in sustaining only routine measles immunization was 3000,^47^ which is particularly important, as several countries are experiencing measles outbreaks concurrent with the pandemic (Fig. 1). The pandemic and ongoing outbreaks pose a substantial threat to immunization programs and are likely to cause additional vaccine-preventable deaths among vulnerable children.

#### Human immunodeficiency virus

At 1.7 million, SSA accounts for approximately 90% of all children living with HIV under 15 years of age,^48^ out of which only 52% have access to treatment.^49^ In 2018, approximately 90% of global AIDS-related deaths under age 20 years occurred in this region.^50^ Sustained viral suppression, especially among children, is dependent on uninterrupted supplies of highly active antiretroviral drugs and robust adherence, often requiring psychosocial support for children and/or caregivers. Pandemic-related movement restrictions and service disruptions are likely to reduce ease of access to HIV treatment services and psychosocial support, leading to poor adherence, deterioration of mental health, and greater HIV-related morbidity and mortality for children.^51^ Interruptions in drug production and supply are further complicated by COVID-19-related regional and international travel restrictions, which also raise concerns about potential HIV drug supply shortages.^52^ In addition, there are concerns that funding and other resources for HIV programs could be diverted to the COVID-19 response.^51^ A modeling analysis by Hogan and colleagues estimates that HIV deaths may increase by up to 10% in the next 5 years due to the impact of the pandemic.^38^

COVID-19 containment measures are also likely to impede access to HIV prevention services, including programs targeting adolescents and prevention of mother-to-child transmission of HIV (PMTCT). The impact of the pandemic on HIV prevention among children is likely to be disproportionately high, as women and children bear the brunt of HIV prevention service gaps in humanitarian emergencies.^53^ For children under 15 years, modeling data suggest that just three months of  PMTCT service disruption could result in new HIV infections spiking by as much as 41% in Mozambique, 53% in Zimbabwe,  70% in Uganda, and 81% in Malawi.^54^

#### Tuberculosis

In 2018, children living in Africa comprised 24% of the estimated 1.1 million children under 15 years with active TB, and 25% of the 230,000 estimated to have died from the disease that year.^55^ These are merely approximations, as many children with active TB go undetected or unreported.^55^ Due to the pandemic, TB prevention and control strategies such as infant Bacillus Calmette–Guérin immunization, community case finding and contact tracing, and directly observed therapy are likely to be disrupted in many countries. Estimates indicate that a 3-month lockdown and 10-month protracted recovery scenario in high TB-burden countries could lead to an additional 10.7% (6.33 million) TB cases and 16% (1.37 million) deaths between 2020 and 2025.^56^ This could translate into an additional ~700,000 cases and ~192,000 deaths from TB in children <15 years old,^55^ a significant proportion of which would occur among African children.

In a recent editorial, child pneumonia experts across the globe warn that, even though COVID-19 in children may be milder as compared to adults, the impact of COVID-19 as a viral pneumonia syndrome may affect children in low- and middle-income settings more severely than those in high-income countries, citing low immunization uptake, severe malnutrition, HIV, and other factors.^57^

#### Sickle cell disease

SCD is a genetic red blood cell disorder with high prevalence among people of African descent, in the Indian subcontinent, and in parts of the Middle East and the Mediterranean region.^58^ SSA accounts for an estimated 79% of ~300,000 infants born annually with SCD worldwide.^59^ The risk of mortality is estimated at 50–90% among infants, and weak health infrastructure often contributes to delays in receiving life-saving interventions, such as pneumococcal vaccination, penicillin prophylaxis, and parental education.^60^ Patients with SCD are considered high risk for COVID-19 and complications due to impaired immunity secondary to functional hyposplenism, increased vulnerability to severe bacterial infections, systemic vasculopathy, and predisposition to thrombosis.^61^ As SARS-CoV-2 affects the respiratory system, it may be difficult to differentiate between symptoms of acute chest syndrome (a manifestation of SCD pulmonary vaso-occlusive disease) and COVID-19 pneumonia. Data on SCD and COVID-19 are limited, and given the high prevalence of SCD in SSA, countries need to proactively optimize access to critical interventions (e.g., oxygen, pain medication, blood supply) while scaling up evidence-based COVID-19 diagnosis and treatment modalities.

#### Co-infections and co-morbidities with SARS-CoV-2

The co-occurrence of SARS-CoV-2 infection with major diseases is likely to exacerbate the impact of the pandemic on health across all ages. Adults with SARS-CoV-2 pneumonia and bacterial superinfection have poorer outcomes,^2,62^ similar to superinfection in influenza and other viral pneumonias.^63^ Data on bacterial superinfection of SARS-CoV-2 pneumonia in children are sparse; available data are largely from small Chinese cohorts describing asymptomatic to moderate illness.^62,64^ There have been no African reports to date.

The gastrointestinal symptoms of COVID-19 may mimic that of rotavirus diarrhea in children.^65^ More common respiratory and gastrointestinal infections present potential challenges in the diagnosis and treatment of COVID-19 pneumonia and diarrhea for children in SSA. There are no published reports on the epidemiology, presentation, or disease course of SARS-CoV-2 and *Plasmodium* spp. co-infection in children. Understanding potential synergistic effects, if any, of SARS-CoV-2-related pulmonary disease and malaria-induced respiratory complications will be critical in preventing associated morbidity and mortality.

Clinical outcomes for children with HIV and COVID-19 co-infections in SSA may be worse than for children elsewhere. The proportion of HIV-infected children with viral suppression in SSA remains lower than the global average,^66^ and these children experience higher rates of HIV-related morbidity and mortality. However, the excess risk posed by COVID-19 to children living with HIV remains unknown. Studies from the US, China, and Spain indicate that the proportion of COVID-19-affected adults with HIV co-infection is <1%.^67–69^ Data from South Africa, which has the largest HIV epidemic globally, suggests that HIV increases the risk of death from COVID-19 by approximately twofold among adults >19 years of age.^70^ Overall, the mortality risk among people living with HIV still appears to be worse among those >50 years and with co-morbidities, including hypertension, obesity/hyperlipidemia, chronic obstructive pulmonary disease, and diabetes.^70,71^

There are also few reports on SCD and COVID 19 co-infection. A US-based case series of 7 patients aged 2–20 years indicated that the majority present with fever, with or without vaso-occlusive episode or acute chest syndrome.^72^ All patients recovered; those hospitalized were treated with hydroxychloroquine and remdesivir, with anakinra prescribed for children with elevated inflammatory markers.^72^

There is currently a lack of reports on COVID-19 and TB or other co-infections among children in SSA or elsewhere, and both observational and interventional data are needed to rapidly fill this critical knowledge gap.^57^

### Social and ethical considerations for children in SSA in the context of the COVID-19 pandemic

#### Domestic, family, and sexual violence and loss of social protections

There have been multiple reports of spikes in domestic, family, and sexual violence following the institution of home isolation and closure of schools and work facilities as COVID-19 containment, and SSA is no exception.^73,74^ Kenya reported a 34% rise in domestic violence, while in South Africa, there was a 37% spike in gender-based violence complaints in the first week of a total lockdown.^73^ Children are often victims and/or witnesses of domestic/family violence, which has a harmful impact on their physical health and mental development and wellbeing.^74,75^ Additionally, as was documented during the Ebola epidemic, school closures and other containment measures result not only in the loss of education but of social protections especially for adolescent girls, leading to consequences such as teenage pregnancy.^73,76,77^ During the 2014–2015 Ebola epidemic in Sierra Leone, teenage pregnancy increased by up to 65% in some communities, secondary to sexual exploitation in the setting of socio-economic hardships.^76,77^ Indeed, with the COVID-19 pandemic, adolescent girls are experiencing significant increases in sexual violence, teen pregnancy, and forced/early marriage across SSA, including in refugee camps.^76^

#### Unintentional injuries

Injuries rank seventh among the top ten health conditions contributing to disability-adjusted life years in children globally.^78,79^ In SSA, the prevalence of injuries is 1,062/100,000 for CU5 and 8,954/100,000 for children 5–14 years; mortality rate is 65 and 33 per 100,000, respectively.^23^ The most common causes of injury-related deaths among SSA children are road traffic accidents, burns, drowning, poisoning, and falls.^80^ In the US and in European countries, COVID-19-related movement restrictions including school closures have significantly reduced the incidence of road traffic accidents^81,82^ and volume of pediatric emergency room visits.^83,84^ However, reports from these settings also indicate that domestic injuries (burns, accidental ingestion) have significantly increased in the same period.^79,84,85^ So far, there is little reported data on the indirect impact of COVID-19 on unintentional child injuries in SSA.

#### Inclusion of children in COVID-19 clinical trials

As of August 8, 2020, a total of 2346 active COVID-19-related trials were registered with Clinicaltrials.gov. Of these, a total of 103 (4.4%) studies were in Africa, with 23 unique studies implemented in SSA (39 in total if counted by country) (Table 2).

**Table 2:** ClinicalTrials.Gov Registry: active COVID-19-related studies in sub-Saharan Africa^a^.

	Country	Total no. of studies	No. of interventional studies (% of all studies)	No. of studies enrolling children aged <18 years (% of all studies)	No. of interventional studies enrolling children aged <18 years (% of studies enrolling children)
1	South Africa	8	6 (75.0)	0 (0.0)	N/A
2	Nigeria	4	3 (75.0)	0 (0.0)	N/A
3	Kenya	3	2 (66.7)	0 (0.0)	N/A
4	Zambia	3	1 (33.3)	0 (0.0)	N/A
5	Zimbabwe	3	1 (33.3)	0 (0.0)	N/A
6	Ghana	2	1 (50.0)	0 (0.0)	N/A
7	Malawi	2	0 (0.0)	0 (0.0)	N/A
8	Mozambique	2	0 (0.0)	0 (0.0)	N/A
9	Sudan	2	1 (50.0)	1 (50.0)^b^	1 (100.0)
10	Tanzania	2	0 (0.0)	0 (0.0)	N/A
11	Botswana	1	0 (0.0)	0 (0.0)	N/A
12	Burkina Faso	1	0 (0.0)	0 (0.0)	N/A
13	Côte d’Ivoire	1	1 (100.0)	0 (0.0)	N/A
14	Democratic Republic of the Congo	1	0 (0.0)	1 (100.0)^c^	0 (0.0)
15	Ethiopia	1	0 (0.0)	1 (100.0)^d^	0 (0.0)
16	Gambia	1	0 (0.0)	0 (0.0)	N/A
17	Senegal	1	1 (100.0)	1 (100.0)^e^	1 (100.0)
18	Uganda	1	1 (100.0)	0 (0.0)	N/A
	TOTAL sub-Saharan Africa	39^f^	18 (46.2)	4 (10.3)	2 (50.0)
	United States of America	502	374 (74.5)	54 (10.7)	24 (44.4)

*N/A* not applicable.

^a^Recruiting and not yet recruiting studies registered at ClinicalTrials.gov as of August 8, 2020.

^b^Sudanese participants aged 5–90 years. Testing oral Gum Arabic as dietary supplement and immune modulator for treatment.

^c^Congolese participants aged 15–75 years. Promoting nutritional supplementation with local foods for COVID-19 patients.

^d^Ethiopian participants of all ages. Profiling immune responses to COVID-19.

^e^Participants aged ≥15 years. Testing safety and efficacy of hydroxychloroquine versus hydroxychloroquine and azithromycin for treatment.

^f^23 unique studies across sub-Saharan Africa; there was no study exclusively targeting children aged <18 years.

Of the 39 SSA studies identified across 18 countries, only 4 (10.3%) include children under 18 years, with just 2 studies targeting children <15 years (Table 2). None of the SSA studies are exclusively enrolling children under 18 years. Counting by country, the number of COVID-19 studies in SSA as a region (*N* = 39) pales in comparison to the number in the US as a country (*N* = 502). The numbers of studies enrolling children (*N* = 2 versus *N* = 24, respectively) are also quite dissimilar.

While severe COVID-19 largely affects adults, children are not spared. There are increasing reports of clusters of a severe multi-system inflammatory syndrome associated with COVID-19 among North American and European children.^86–89^ Our understanding of this presentation is limited but rapidly evolving. It is not yet known what impact this sequela of SARS-CoV-2 infection may have on viral, bacterial, or parasitic co-infections. The Africa Centers for Disease Control and Prevention has issued a health advisory for this syndrome in children; however, there are currently no available data among African children.^16^

The exclusion of children from COVID-19 drug and vaccine trials relegates the health and wellbeing of children to reliance on adult safety and efficacy data, with potentially unpredictable and detrimental effects.^90^ Furthermore, given the significant impact of genetics and HIV status on immune ontogeny and function, which in turn inform vaccine design, it is critical to include African children, many of whom are infected with HIV, in vaccine trials.^91^

## Discussion

In addition to advancing the body of knowledge and response to COVID-19 in children, the indirect effects of the pandemic must be identified and concurrently addressed. For children in SSA, it will be important to optimize case detection and prompt management of highly prevalent diseases, such as malaria, HIV, and TB, and minimize interruptions for those on long-term treatment.^36,92,93^ Other infectious disease prevention through established national immunization programs^94–96^ must be sustained, while scaling up access to accurate diagnostics and care for SARS-CoV-2 and other causes of undifferentiated febrile illness.^97,98^ High-risk children with NCDs such as undernutrition and SCD must be targeted for sustained immunization and access to other critical interventions. The WHO’s Action Plan on Child Wasting and African Union’s Nutrition Strategy supports strengthening of national food, health, and social protection programs.^99,100^ However, this emergency pandemic situation requires promptly implemented palliatives of food and financial support that prioritize the most vulnerable children and families.^101^

Pandemic-responsive plans are being rolled out to minimize the negative impact of COVID-19 on targets set for HIV,^93^ TB,^92^ and malaria^36,102^ in SSA countries. Donor agencies have instituted measures to mitigate the effect of the pandemic on major diseases, especially in resource limited in settings. The Global Fund to Fight AIDS, Tuberculosis and Malaria has created a new funding mechanism that dedicates at least $500 million to fighting COVID-19 in hard-hit countries^103^ and is also reallocating portions of previously disbursed funds for the COVID-19 response, including epidemic preparedness assessment, laboratory testing, surveillance infrastructure, infection control in health facilities, and information campaigns.^104^ The Global Fund also provides regularly updated online information on how COVID-19 is affecting the global response to HIV, TB, and malaria.^105^

The US President’s Emergency Plan for AIDS Relief is decentralizing HIV services nearer to patient homes during movement restrictions, through strategies like community drug delivery.^106^ To reduce HIV-infected or -affected children’s exposure to COVID-19, caregivers have been advised to access facility-based services without their wards. Remote case management and support is to be prioritized for vulnerable children, including those with treatment failure and particularly severe psychosocial challenges.^106^ Implementing partners have been sensitized to potential spikes in gender-based violence and sexual exposure to HIV and are strengthening programming for prevention and survivor care. Furthermore, where feasible, key primary services and ancillary care such as adolescent support groups and adherence counseling are being migrated to social media applications.^106^

Telehealth (also known as telemedicine) allows for continued but remote healthcare delivery during movement restrictions and isolation.^107^ Telehealth infrastructure in SSA is relatively undeveloped; however, social media and mobile health applications are being leveraged to facilitate interactive provider–patient consultations during the pandemic.^108–110^ South Africa is one of the few SSA countries to have telehealth guidelines, and these 2014 guidelines have been updated specifically for COVID-19.^111^ Much is left to be addressed for telehealth in SSA, such as accessibility and affordability for healthcare facilities, providers and patients, documentation and billing, patient privacy, and other regulatory issues.

Public health responses to the pandemic in SSA countries are evolving; however, attention to social determinants of health is sadly inadequate. Measures such as expanded access to courts, legal protection, and housing to address the needs of vulnerable children should be instituted by governments as an ethical imperative.^112^ Furthermore, civil society organizations, health personnel, researchers, and other relevant stakeholders need to collectively ensure the safety and protection of children, especially during this pandemic.^73^

Studies from outside Africa have highlighted the disproportionate impact of COVID-19 on different subpopulations, including people of color.^113–115^ In order to alleviate immediate and long-term harmful effects, we need evidence on the extent to which social determinants of health such as poverty, physical environment, gender, and racial/ethnic discrimination are affecting children in SSA due to the pandemic.

While pediatric numbers for COVID-19 may be assumed small, the dearth of data in SSA countries limits meaningful study for an appropriate public health response for children. The under-inclusion of SSA children in clinical trials further limits the safe and efficacious use of new and/or repurposed drugs and vaccines for COVID-19 for this population. There is also sub-optimal understanding on the role of children in community transmission of SARS-CoV-2 in SSA. So far, tracing and testing in SSA is largely focused on adults.^73^

Conflict and post-conflict areas will be more likely to face data gaps, and more extensively so. Countries experiencing intense conflict and forced displacement (e.g., Central African Republic, the Democratic Republic of the Congo, Nigeria, Somalia, South Sudan, and Sudan) are expected to be at particularly high risk for COVID-19 transmission and deaths.^116^ Due to additional safety concerns and health service disruptions, these countries are likely to experience greater strains in their testing/reporting capacity and thus may have significant undercounting of cases secondary to limited disease surveillance.^116^ Poor COVID-19 data can be strengthened through strategic sentinel surveillance to inform tailored responses for conflict/post-conflict settings.^117^

## Conclusion

The COVID-19 pandemic is threatening efforts to prevent and control the major causes of child morbidity and mortality in SSA. As long as this pandemic persists, and even in its aftermath, its ripple effects will impact on children’s health, whether or not they are ever infected by SARS-CoV-2. These effects will take an especially heavy toll on children in SSA. However, this pandemic presents an opportunity to accelerate both targeted (testing laboratories and infectious disease treatment centers) and comprehensive/cross-cutting action (e.g., social support, policy changes, and new funding streams). The changes should focus not only on *what* can or should be done but also on *how* to do things differently for sustainable impact in these rapidly changing circumstances. Even as the COVID-19 pandemic continues, things should not fall apart for children in SSA.
